# COVID-19 vaccine hesitancy: assessing the prevalence, predictors, and effectiveness of a community pharmacy based counseling intervention

**DOI:** 10.1186/s12889-023-17532-4

**Published:** 2024-01-06

**Authors:** Joshua Kiptoo, John Isiiko, Tadele Mekuriya Yadesa, Tumugumye Rhodah, Paul E. Alele, Edgar Mugema Mulogo

**Affiliations:** 1 Department of Pharmacy, Faculty of Medicine, Mbarara University of Science and Technology, Mbarara, Uganda; 2 Department of Pharmacy, Uganda Cancer Institute, Mbarara, Uganda; 3 Department of Clinical Pharmacy and Pharmacy Practice, Kampala International University, Ishaka, Uganda; 4 Department of Nursing, Mbarara Regional Referral Hospital, Mbarara, Uganda; 5 Department of Pharmacology and Therapeutics, Faculty of Medicine, Mbarara University of Science and Technology, Mbarara, Uganda; 6 Department of Community Health, Faculty of Medicine, Mbarara University of Science and Technology, Mbarara, Uganda

**Keywords:** COVID-19, Vaccine, Hesitancy, Acceptance, Pharmacy, Intervention, Counseling, Communication, Uganda

## Abstract

**Background:**

Coronavirus disease (COVID-19) vaccine hesitancy is a global challenge. In low- and middle-income countries (LMICs), the problem has persisted despite vaccine availability and decreasing infections. In Uganda, there is still limited information on the extent and predictors of vaccine hesitancy. This study sought to assess the prevalence and predictors of COVID-19 vaccine hesitancy, and the effectiveness of an intervention that involved community pharmacy counseling in combating COVID-19 vaccine hesitancy.

**Methods:**

A total of 394 participants were enrolled in a 4-week prospective cohort interventional study. The study was conducted across eight community pharmacies in Mbarara City, between 9:00 AM and 5:00 PM daily. The study personnel ascertained the vaccination status of all clients seeking community pharmacy services. All unvaccinated clients were consecutively assessed for eligibility, and eligible clients were systematically enrolled after receiving the community pharmacy services for which they requested. The study intervention involved structured participant counseling (within the pharmacy premise), follow-up short message service (weekly), and telephone calls (bi-weekly). Only participants who did not accept to receive the COVID-19 vaccine despite counseling were followed up for four weeks, or until they accepted to receive a COVID-19 vaccine. The effectiveness of the community pharmacy counseling intervention was determined as an increase in COVID-19 vaccine acceptance, and desirable attitudinal change towards COVID-19 disease, vaccination exercise, and vaccines. Descriptive analysis was used to summarize data, and multivariate analysis was used to determine the predictors of COVID-19 vaccine hesitancy. A *p*-value < 0.05 was considered statistically significant.

**Results:**

Out of 394 participants, 221 (56%) were hesitant to receive a COVID-19 vaccine. Participants expressed several reasons (mean 2±1) for COVID-19 vaccine hesitancy, mostly concerning vaccine safety (*N*=160, 47.3%). The overall COVID-19 vaccine acceptance rate increased by 25.4 percent points (43.9 – 69.3 percent points) after the study intervention. Age, religion, level of education, distance from the nearest public health facility, having a friend/family diagnosed with COVID-19, and personal suspicion of contracting COVID-19 were significant predictors of COVID-19 vaccine hesitancy.

**Conclusion:**

COVID-19 vaccine hesitancy is a big challenge in Uganda. A mix of sociodemographic and COVID-19 vaccine perceptions are the key predictors of COVID-19 vaccine hesitancy. Although COVID-19 vaccines were not available at the time of the study, this study found that structured counseling interventions can improve COVID-19 vaccine acceptance rates. Larger prospective studies should evaluate the effectiveness of similar interventions in community pharmacies and other healthcare settings.

## Introduction

The COVID-19 pandemic exposed the vulnerability of global health systems, especially in low-income countries [1–3]. Although the COVID-19 disease burden drastically declined due to preventive measures including quarantine, lockdown, and infection prevention control (IPC) [4–6], these measures remain inadequate in curbing the COVID-19 pandemic. The global socio-economic destabilization that was triggered by the pandemic continues to haunt the world [7–12]. Concurrently, adherence to COVID-19 IPC measures remained generally low throughout the pandemic [8, 13–15]. Therefore, regardless of global concerns about equitable access to COVID-19 vaccines [16–18], COVID-19 vaccine hesitancy has been recognized as a considerable deterrent to multi-sectoral COVID-19 vaccine decentralization efforts [19–24]. The WHO defines vaccine hesitancy as the “delay in acceptance or refusal of vaccines despite availability of vaccination services” [21].

Vaccine hesitancy is an age-old phenomenon [25], that has persisted despite the historical role of vaccines in mitigating infections of public health concerns [26]. The invention of COVID-19 vaccines was commendable [27, 28], as it significantly reduced disease burden and accelerated herd immunity against SARS-COV-2 variants [11, 29–31]. The prevalence of COVID-19 vaccine hesitancy is between 23.6 and 97% globally [20, 32], and about 42.2% in Uganda [33]. In Uganda, five approved vaccines against the common SARS-CoV-2 variants are available, including; Spikevax (Moderna), Cormirnaty (Pfizer/BioNTech), Janssen (Johnson & Johnson), Vaxzeviria (Oxford/AstraZeneca), and CoronaVac (Sinovac) [34]. However, similar to global trends, COVID-19 vaccine uptake remains low [35, 36]. Because vaccine-induced herd immunity requires vaccination of a critical population mass [37], vaccine hesitancy is a significant contributor to the low COVID-19 vaccine uptake [20, 32, 35, 36].

To achieve the national vaccination targets, integrated interventional strategies are necessary [38]. The “3Cs” model [25] is among the theories advanced to explain the complex interaction of factors relating to an individual’s acceptance or hesitancy towards COVID-19 vaccines. The “3C” behavioral model suggests that adoption of health services is driven by a complex interaction of Complacency, Convenience, and Confidence in the public [39–41]. For example, concerns related to vaccine hesitancy broadly regard national vaccination programs, whereas others are specific to the context of COVID-19 and the available vaccines [39, 42, 43]. Other established factors include risk perception of harm from the virus [44], mistrust of vaccine manufacturers [45], and credibility of the vaccine development process [46]. Implying that COVID-19 mis- and disinformation may have contributed to the prevalent problem of COVID-19 vaccine hesitancy [47, 48]. This study therefore, is based on the “3Cs” behavioral model [25], which identifies communication as a tool and less of a determinant for successful immunization [49]. Counseling and education interventions, including telephone calls and Short Message Service (SMS) reminders, have been reported to effectively dispel fear and anxieties arising from misinformation about the COVID-19 vaccines [11, 50, 51]. In this study, we sought to determine the prevalence and predictors of COVID-19 vaccine hesitancy, as well as the effectiveness of a community pharmacy based counseling intervention.

## Methods and materials

### Study design and setting

We conducted a prospective interventional study in community pharmacies within Mbarara city, between September 1 and November 30, 2022. Mbarara City is located in Mbarara district, 266 km southwest of Kampala, which is the capital city of Uganda. The current national census report indicates a total population of 195,318 within Mbarara city and 472,625 in Mbarara district. We randomly selected 8 out of 86 licensed drug outlets in Mbarara city [52]. The 8 community pharmacies majorly stock and avail drugs to the general public under the supervision of a duly licensed pharmacist.

### Study population, sample size, and sampling technique

We targeted all unvaccinated clients seeking community pharmacy service. All potential participants ages ≥ 18 years and consenting to participate in the study were included in the study. Participants without a mobile phone, verifiable telephone contact or residence at the time of enrolment were excluded from the study. To determine the study sample size (n), we used the single population proportion formula. Based on a nationally representative survey, a COVID-19 vaccine hesitancy prevalence of 57.8% was considered (95% CI and 5% margin of error) [33]. A calculated sample size of 375 was considered. Considering a 10% non-response rate, the target sample size was 413. A Microsoft Excel 2020 random number generator was used to randomly select the 8 pharmacies from among the 86 licensed drug outlets in Mbarara city. Eligible participants were selected using a systematic random sampling method. Based on the previous month’s records, the total number of clients in all the pharmacies in a month was estimated to be 5,280. A sampling interval of 12 was determined by dividing the estimated target population by the estimated sample size of 413. Thus, during the enrolment duration of 4 weeks in each of the 8 pharmacies, every twelfth client was enrolled until the intended sample size was achieved.

### Study Intervention

#### Research team

The intervention team consisted of 3 clinical pharmacists and 8 research assistants. The research assistants underwent extensive skills and knowledge pre-training regarding the study protocol, safety and effectiveness of the WHO approved COVID-19 vaccines currently available at the local vaccination centers in Uganda, and the key counseling points and ethical considerations on conducting research regarding COVID-19 vaccines and vaccination.

#### Intervention

The study intervention involved a counseling service conducted within the community pharmacy premise, and follow-up communication intervention. Communication intervention comprised of weekly short message service (SMS) and bi-weekly telephone call post enrolment.

#### Recruitment of participants for the intervention

The need for counseling and attitudinal assessment differed among study participants, based on the individual participant’s willingness to vaccinate and the reasons for vaccine hesitancy. Counseling was tailored to the individual participant’s reasons for COVID-19 vaccine hesitancy. Except for participants who were willing to receive a COVID-19 vaccine at first inquiry, counseling and attitudinal assessment were done for all participants who were not willing to be vaccinated at first questioning within the pharmacy. Only study participants who expressed interest in being vaccinated after counseling in the study site were assessed for pre- and post-intervention attitudinal assessment, whereas those who remained hesitant to receive a COVID-19 vaccine despite counseling were not assessed for a repeat attitudinal change using the collapsed 3-point Likert scale. Participant follow-up was done for 4 weeks post enrolment, among participants who remained hesitant to receive the COVID-19 vaccine despite the counseling. During the follow-up communication, participants received a customized weekly short message service (SMS), and a bi-weekly telephone call. The SMS provided reminder information regarding the benefits of COVID-19 vaccination and the availability of COVID-19 vaccines at the designated national COVID-19 vaccination centers within Mbarara city. A telephone call was only made after successful delivery of the SMS to the study participant. During the phone call, participants were counseled if they had any new concerns regarding COVID-19 vaccines. All study participants were followed up until either they accepted to receive a COVID-19 vaccine, or at the end of the study period in the fourth week. The effectiveness of the intervention was pre-determined as an increase in the vaccine acceptance rate, as well as a statistically significant difference in the pre- and post-intervention participants’ mean Likert scores in participant responses.

### Study variables

The independent variables collected included; socio-demographic characteristics (age, marital status, education level, residence, occupation, and distance from nearest health facility), COVID-19 disease related factors (family/friend diagnosed with COVID-19, illness with chronic disease, suspicion of having COVID-19), and participant attitude towards COVID-19 disease, COVID-19 vaccination programs, and COVID-19 vaccines. COVID-19 vaccine hesitancy was the measured dependent variable.

### Data collection

An interviewer-administered questionnaire was iteratively developed and used it to obtain the participant’s demographics and health-related data. Based on expert knowledge of study investigators, and the “3Cs” Behavioral Model of determinants of COVID-19 vaccine hesitancy [25]; a structured interview guide was developed and divided into three thematic areas, i.e., concerns regarding COVID-19 disease, COVID-19 vaccination exercise, and COVID-19 vaccines. We used a collapsed 3-point Likert scale to assess participant attitudes in the three thematic areas, before and after counseling [53]. Individual participant Likert scores (responses) were categorized into three categories, i.e., Strongly Disagree/Disagree (SD/D) (scores ≤1), Neutral/Don’t know (N) (score = 2), and Strongly Agree/Agree (SA/A) (scores 3 – 4). Patients were then followed up for further assessment for COVID-19 vaccine acceptance. During follow-up telephone calls, a structured questionnaire was used to determine the participant’s acceptance of COVID-19 vaccines. All study participants were interviewed only after they received the community pharmacy service they intended to get. Participants were then interviewed/counseled in a separate space or room within the pharmacy premises for a period not exceeding 10 minutes. Only participants who were hesitant to receive the COVID-19 vaccines were asked to provide reasons where they were hesitant to receive the vaccines. Based on the participant’s attitude and concerns about the COVID-19 vaccines, counseling was provided tailored to the participant’s reasons for COVID-19 vaccine hesitancy. A pre- and post-counseling measurement of the participant’s attitude was done based on the participant’s willingness to vaccinate at the first inquiry. Participant follow-up was done as described in the ‘intervention’ section.

### Statistical analysis

Data were entered into a Microsoft Excel book sheet, and later analyzed using Statistical Package for Social Sciences software (SPSS version 21, SPSS Inc., Chicago, IL, USA). Descriptive analyses were used to summarize socio-demographic and clinical information, vaccine acceptance, reasons for vaccine hesitancy, and changes in attitude towards COVID-19 disease, vaccination and vaccines. A paired samples t-test was used to determine the mean difference between the pre- and post- intervention Likert scores. Binary logistic regression analysis was used to determine the predictors of COVID-19 vaccine hesitancy. Only variables with *p*-value < 0.25 at univariate level, were included in the final multivariate logistic regression model for analysis. Odds ratios were estimated at a 95% confidence interval, and *p*-value < 0.05 was considered statistically significant in multivariate logistic regression.

### Ethical considerations

The study was approved by the Mbarara University of Science and Technology Research Ethics Committee (number: MUST-2022-567). Administrative clearance was obtained from the City clerk, Mbarara City (CR/MC/331) to conduct research in community pharmacies. Permission was sought from the general manager of each community pharmacy prior to the study commencement. All participant information was stored on password-protected computers, only accessible by the investigators. Informed written consent was obtained from each eligible pharmacy participants prior to recruitment.

## Results

### Background characteristics

The majority of the study participants were aged 18-35 years (mean; 29.5±10.2 years), identified as male (*N*=247, 62.7%), and were single (*N*=230, 58.4%). Most participants lived in semi-rural/rural areas (*N*=255, 64.7%) and had attained at least a secondary education (*N*=284, 72.1%) (Table 1).

**Table 1:** Sociodemographic factors Versus COVID-19 vaccine hesitancy status among clients visiting community pharmacies in Mbarara city, southwestern Uganda

**Variables**	**Categories**	**Vaccine hesitant** **n (%)**		**Frequency n (%)**
		**No**	**Yes**	
Age (years)	Youth (≤ 25)	88 (49.2)	91 (50.8)	179 (45.4)
	Young Adults (26-35)	47 (37.9)	77 (62.1)	124(30.5)
	Adults (≥ 36)	38 (41.8)	53 (58.2)	91 (23.1)
Gender	Female	69 (46.9)	78 (53.1)	147 (37.3)
	Male	104 (42.1)	143 (57.9)	247 (62.7)
Marital status	Single	100 (43.5)	130 (56.5)	230 (58.4)
	Married	61 (43.0)	(57.0)	142 (36.0)
	Divorced Or Widowed	12 (54.5)	10 (45.5)	22 (5.6)
Residence	Urban	60 (43.5)	78 (56.5)	138 (35.3)
	Semi-Urban/Rural	113 (44.3)	142 (55.7)	255 (64.7)
Religion	Catholic	66 (41.8)	92 (58.2)	158 (40.1)
	Anglican/Protestant	74 48.1)	80 (51.9)	154 (39.1)
	Muslim	22 (56.4)	17 (43.6)	39 (9.9)
	Pentecostal	2 (10.0)	18 (90.0)	20 (5.1)
	Others	9 (39.1)	14 (60.9)	23 (5.8)
Level of education	No Formal Education	0 (0.0)	9 (100.0)	9 (2.3)
	Primary	64 (63.4)	37 (36.6)	101 (25.6)
	Secondary	73 (49.0)	76 (51.0)	149 (37.8)
	Tertiary	36 (26.7)	99 (73.3)	135 (34.3)
Occupation	Unemployed	23 (48.9)	24 (51.1)	47 (11.9)
	Student	12 (27.3)	32 (72.7)	44 (11.2)
	Peasant	25 (51.0)	24 (49.0)	49 (12.4)
	Science/Medical/Health	8 (25.0)	24 (75.0)	32 (8.1)
	Business Enterprise	46 (58.2)	33 941.8)	79 (20.1)
	Transportation	27 (51.9)	25 (48.1)	52 (13.2)
	Other	32 (35.2)	59 (64.8)	91 (23.1)
Distance from nearest public health facility (km)	Near (≤ 1)	117 (49.6)	119 (50.4)	236 (59.9)
	Far (> 1)	56 (35.4)	102 (64.6)	158 (40.1)

Almost all the study participants had access to COVID-19 related information (*N*=368, 93.7%), mainy through radio/television (*N*=223, 60.4%). Most participants were not aware of a friend or relative who had died of COVID-19 related complication (*N*=303, 76.9%). In addition, most participants had not taken any vaccine other than COVID-19 vaccines in their adult life (*N*=261, 66.2%), neither did they have a personal suspicion of contracting COVID-19 disease in the near past or during the study period (*N*=241, 61.2%). Lastly, most participants had not been diagnosed with a chronic disease in the near past (*N*=241, 61.2%) (Table 2).

**Table 2:** Participant COVID-19 experiences versus COVID-19 vaccine hesitancy status

**Variables**	**Categories**	**Vaccine hesitant, n (%)**		**Frequency (%)**
		**No**	**Yes**	
Access to information regarding COVID-19 disease and vaccines	No	10 (41.7)	14 (58.3)	24 (6.3)
	Yes	163 (44.3)	205 (55.7)	368 (93.7)
Channels of information access about COVID-19, vaccines and vaccination programs	Radio/TV	115 (51.6)	108 (48.4)	223 (60.4)
	Social media	13 (39.4)	20 (60.6)	33 (8.9)
	Friends/peers	2 (66.7)	1 (33.3)	3 (0.8)
	Newspaper	2 (100.0)	0 (0.0)	2 (0.5)
	≥ 1 source	30 (27.8)	78 (72.2)	108 (29.3)
Friend or relative diagnosed with COVID-19	No	103 (51.5)	97 (48.5)	200 (50.8)
	Yes	70 (36.1)	124 (63.9)	194 (49.2)
Friend or relative died of COVID-19 associated complications in the past or present	No	138 (45.5)	165 (54.5)	303 (76.9)
	Yes	35 (38.5)	56 (61.5)	91 (23.1)
Received other vaccine(s) other than COVID-19 vaccines after age of 18?	No	116 (44.4)	145 (55.6)	261 (66.2)
	Yes	57 (46.0)	76 (54.0)	124 (33.8)
Has a chronic disease	No	151 (44.8)	186 (55.2)	337 (85.5)
	Yes	22 (38.6)	35 (61.4)	57 (16.5)
Personal suspicion to have acquired COVID-19 disease at any time	No	114 (47.3)	127 (52.7)	241 (61.2)
	Yes	59 (38.6)	94 (61.4)	153 (38.8)

### Primary outcome: prevalence of COVID-19 vaccine hesitancy

**Fig. 1:**
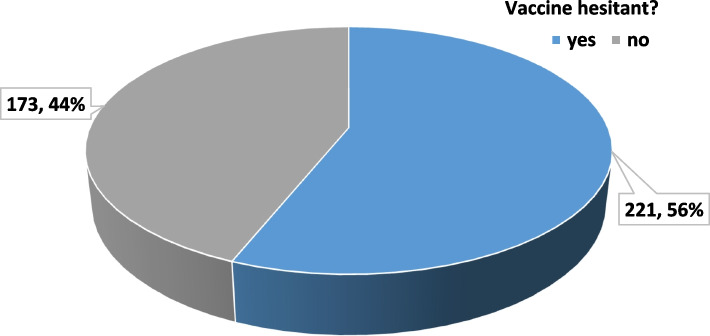
Proportion of study participants hesitant to receive a COVID-19 vaccine

Vaccine hesitancy was defined as participant unwillingness to receive a COVID-19 vaccine at the time of interview by the research assistant, irrespective of the participant’s change of mind and willingness to receive a COVID-19 vaccine post intervention. Out of the 394 participants interviewed, over half (221, 56.0%) were hesitant to receive a COVID-19 vaccine (Fig. 1).

### Secondary outcome: determine the reasons for vaccine hesitancy

Reasons for COVID-19 vaccine hesitancy were only determined among respondents who were hesitant to receive the COVID-19 vaccine at first inquiry.

**Fig. 2:**
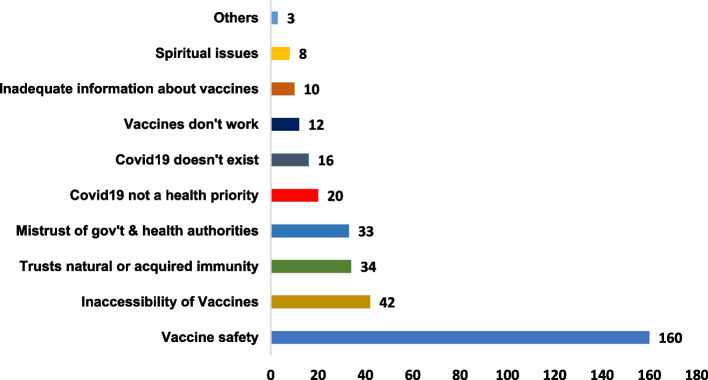
Reasons for hesitancy to receive a COVID-19 vaccine

The reasons for vaccine hesitancy were obtained through open-ended questions and documented in the questionnaire. Among the 221 study participants who were hesitant to receive a COVID-19 vaccine; 338 (Mean ± SD = 2±1) reasons for vaccines hesitancy were documented. The most prevalent reasons for COVID-19 vaccine hesitancy included; vaccine safety (*N*=160, 47.3%), inaccessibility of vaccines (*N*=42, 12.4%), trust in natural or acquired immunity against COVID-19 (*N*=34, 10.1%), mistrust in government or related health authorities (33,9.8%), COVID-19 not being an urgent health priority (*N*=20, 5.9%), and COVID-19 being non-exist (*N*=16, 4.7%) (Fig. 2).

### Tertiary objective: predictors of COVID-19 vaccine hesitancy

All independent variables with *p*-value < 0.05 in the univariate analysis were subjected to a multivariate logistic regression analysis. In multivariate analysis, being an adult (age, ≥ 35 years)(COR = 2.03 [1.05, 3.90 at 95% CI]; *p*-value = 0.035), identifying as a Pentecostal Christian (COR = 8.59 [1.63, 45.25 at 95% CI]; *p*-value = 0.011), maximum level of education being primary (COR = 0.16 [0.07, 0.48 at 95% CI]; *p*-value = 0.000), residing far away from a health facility (> 1 km)(COR = 1.77 [1.10, 2.86 at 95% CI]; *p*-value = 0.019), having a friend/relative diagnosed with COVID-19 (COR = 1.64 [1.01, 2.66 at 95% CI]; *p*-value = 0.046), or having a personal suspicion to have been infected with COVID-19 (COR = 1.66 [1.01, 2.73 at 95% CI]; *p*-value = 0.047) were determined to be statistically associated with COVID-19 vaccine hesitancy (Table 3).

**Table 3:** Predictors of COVID-19 vaccine hesitancy among clients visiting community pharmacies in Mbarara city, southwestern Uganda

**Variable**	**Categories**	**COR (95% CI)**	***p*-value**	**AOR (95% CI)**	***p*-value**
Age (years)	18 – 25	1		1	
	26 – 35	1.03 (0.77, 1.39)	0.823	1.72 (0.96, 3.08)	0.069
	>= 35 (adults)	1.64 (1.14, 2.36)	0.008^*^	2.03 (1.05, 3.90)	0.035^*^
Religion	catholic	1		1	
	Anglican Or protestant	1.08 (0.79, 1.48)	0.629	0.81 (0.47, 1.37)	0.427
	Muslim	0.77 (0.41, 1.46)	0.425	0.88 (0.39, 1.98)	0.756
	Pentecostal	9.00 (2.01, 38.79)	0.003^*^	8.59(1.63, 45.25)	0.011^*^
	other	1.56 (0.67, 3.59)	0.301	1.20 (0.43, 3.33)	0.723
Level of formal education	None	1		1	
	Primary	0.58 (0.39, 0.87)	0.008^*^	0.16 (0.07, 0.48)	0.000^*^
	Secondary	1.04 (0.76, 1.44)	0.806	0.35 (0.16, 0.76)	0.070
	tertiary	2.75 (1.88, 4.03)	0.000^*^	0.56 (0.22, 1.40)	0.213
Occupation	None	1		1	
	Student	2.67 (1.37, 5.18)	0.004^*^	2.63 (0.96, 7.24)	0.060
	Peasant	0.96 (0.55, 1.68)	0.886	0.86 (0.34, 2.18)	0.749
	Science/health/medical	3.00 (1.35, 6.68)	0.007^*^	2.48 (0.76, 8.11)	0.134
	Business	0.717 (0.46, 1.12)	0.145	0.78 (0.35, 1.74)	0.550
	transportation	0.93 (0.54, 1.60)	0.782	1.39 (0.58, 3.35)	0.466
	other	1.84 (1.20, 2.84)	0.005^*^	2.18 (1.00, 4.77)	0.051
Distance from nearest health facility (km)	=< 1 (near)	1		1	
	> 1 (far)	1.82 (1.32, 2.52)	0.000^*^	1.77 (1.10, 2.86)	0.019*
Friend/relative diagnosed with COVID-19	no	1		1	
	yes	1.77 (1.23, 2.38)	0.000^*^	1.64 (1.01, 2.66)	0.046^*^
Personal suspicion of having COVID-19 disease?	no	1		1	
	yes	1.59 (1.15, 2.21)	0.005^*^	1.66 (1.01, 2.73)	0.047^*^

*COR* Crude Odds Ratio, *AOR* Adjusted Odds Ratio. *p-value <0.05

### Tertiary objective: effectiveness of a community pharmacy based counseling intervention towards COVID-19 vaccine acceptance

The COVID-19 vaccine acceptance rate was only assessed before and after the study intervention. The vaccine acceptance rate increased by 25.4 percent points by the end of the study intervention.

**Fig. 3:**
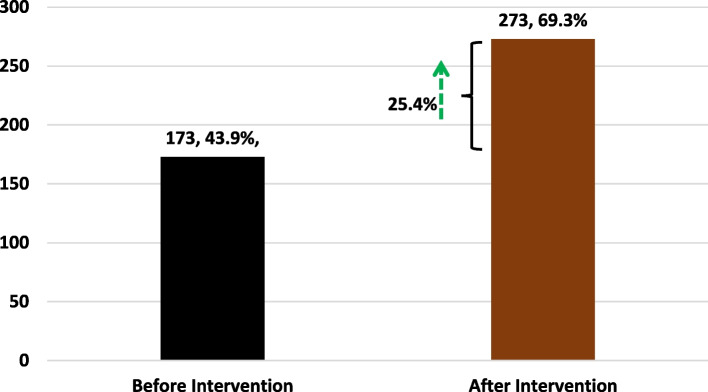
COVID-19 vaccine acceptance rate before and after study intervention

Out of the 394 study participant interviewed, the pre-intervention (baseline) COVID-19 vaccine acceptance rate was 43.9% (173/394). At the end of the study period (fourth week), the number of study participants willing to receive the COVID-19 vaccine had increased to 273, representing a post intervention COVID-19 vaccine acceptance rate of 69.3% (273/394). Therefore, the difference of the pre- and post- intervention COVID-19 vaccine acceptance rate was 25.4 percent points (Fig. 3).

### Attitudinal change using a collapsed 3-point Likert scale

Overall, there was a statistically significant difference in the pre- and post- intervention Likert scores in 74.1% [20] of the questions assessing attitude towards COVID-19 disease, vaccination exercise, and vaccines (*p* value < 0.05) (Table 4) and (Table 5)*.*

**Table 4:** COVID-19 disease and vaccination exercise: pair mean difference (Likert scores) before and after study intervention

	**S/N**	**Question**	**Paired Mean Differences**			***p*-value**
			**Mean score** **(pre-post)**	**SD**	**95% CI**	
**COVID-19 disease**	1.	COVID-19 can cause a serious disease	-0.18	0.59	-0.29, -0.07	0.002^*^
	2.	Not everyone is at risk of contracting COVID-19	0.27	0.87	0.10, 0.44	0.002^*^
	3.	COVID-19 can kill	0.15	0.74	0.01, 0.30	0.038^*^
	4.	COVID-19 is not preventable	0.10	0.64	-0.03, 0.22	0.132
	5.	COVID-19 easily spread by touching infected surfaces	-0.08	0.45	-0.16, 0.01	0.088
	6.	COVID-19 is not spread by being closer/shaking hands with infected people	0.09	0.42	0.01, 0.17	0.038^*^
	7.	COVID-19 is no longer present in our community	0.26	0.88	0.09, 0.43	0.003^*^
**COVID-19 Vaccination exercise**	8.	Vaccination is effective in controlling COVID-19	-0.31	0.63	-0.43, -0.19	0.000^*^
	9.	Everyone should be vaccinated against COVID-19	-0.39	0.76	-0.53, -0.24	0.000^*^
	10.	It is not good that MoH recommended COVID-19 vaccines for all adults	-0.22	0.63	-0.34, -0.10	0.000^*^
	11.	I'm not convinced by MoH advocacy for COVID-19 vaccines	-0.05	0.57	-0.16, 0.06	0.401
	12.	Family/friends play an important role in persuading me to vaccinate against COVID-19	-0.15	0.51	-0.25, -0.05	0.003^*^
	13.	I will not incur any expenses to receive COVID-19 vaccine	0.12	0.75	-0.02, 0.26	0.096
	14.	Vaccines may not be readily available at my nearest health facilities	-0.12	0.59	-0.23, -0.01	0.037^*^
	15.	There is no need to get vaccinated especially after having COVID-19	0.50	0.88	0.33, 0.67	0.000^*^
	16.	I would prefer to take my COVID-19 vaccine in pharmacies than public health facilities.	-0.20	0.58	-0.31, -0.09	0.000^*^
	17.	COVID-19 vaccination process can impose other health risks to me	0.08	0.67	-0.05, 0.21	0.200

*COR* Crude Odds Ratio, *AOR* Adjusted Odds Ratio. *P-value <0.05. *SD* Standard deviation

**Table 5:** COVID-19 vaccines: pair mean difference (Likert scores) before and after study intervention

	**S/N**	**Question**	**Paired Mean Differences**			
			**Mean score (pre-post)**	**SD**	**95% CI**	***p*-value**
**COVID-19 Vaccines**	1.	COVID-19 vaccines are generally safe for use	-0.50	0.73	-0.63, -0.36	0.000^*^
	2.	COVID-19 vaccines can cause harmful effects	-0.08	0.51	-0.18, 0.02	0.095
	3.	COVID-19 vaccines never cause life threatening effects	0.13	0.71	-0.01, 0.26	0.061
	4.	Safety of different vaccines in the market differ	-0.15	0.71	-0.28, -0.01	0.032^*^
	5.	COVID-19 vaccines reliably protect us from COVID-19	-0.30	0.67	-0.43, -0.18	0.000^*^
	6.	It is safe to mix different COVID-19 vaccines	-0.31	0.84	-0.47, -0.15	0.000^*^
	7.	All types of COVID-19 vaccines are equally protective	-0.18	0.61	-0.30, -0.07	0.002^*^
	8.	It's important to complete all doses of approved COVID-19 vaccines	-0.11	0.53	-0.21, -0.01	0.033^*^
	9.	I can get COVID-19 from COVID-19 vaccines	0.28	0.69	0.14, 0.41	0.000^*^
	10.	I have to complete the remaining COVID-19 vaccine doses after getting minor side effects	-0.42	0.75	-0.56, -0.28	0.000^*^

*COR* Crude Odds Ratio, *AOR* Adjusted Odds Ratio. *P-value <0.05. *SD* Standard deviation

## Discussion

The goal of this study was to explore the prevalence and predictors of hesitancy towards COVID-19 vaccines among community-dwelling adults in Mbarara City, Southwestern Uganda. The study also determined the effectiveness of a counseling intervention delivered in a community pharmacy setting. The level of hesitancy to COVID-19 vaccines was found to be high (56%) (Fig .1). The majority (*N*=236, 70%) of reasons for hesitancy to receive COVID-9 vaccines, were related to either the safety of vaccines (47.3%), inaccessibility of vaccines (12.4%), or individual trust in individual immunity against COVID-19 disease (10.1%) (Fig. 2). Generally, a community pharmacy based counseling intervention increased the COVID-19 vaccine acceptance rate by 25.4 percent points (43.4% - 69.3%) (Fig. 3), and positively changed participants’ attitude towards COVID-19 (Tables 4, 5). Age, religion, education level, distance from the nearest public health facility, having a friend/relative diagnosed with COVID-19, and personal suspicion of having contracted COVID-19 within the past 2 years, were statistically significant predictors of COVID-19 vaccine hesitancy.

More than half (56%) of the population in our setting was hesitant to receive the COVID-19 vaccines (Fig. 1). A findings that does not differ from the average global prevalence of COVID-19 vaccine hesitancy [32], as well as studies from rural communities in Ethiopia and India [54, 55]. However, our prevalence is higher than previous published surveys that reported less than 30% prevalence of COVID-19 vaccine hesitancy in Uganda [41, 43]. The study by *Bongomin et al*. focused on high-risk populations, including older adults and multimorbid individuals, with documented vaccine hesitancy, albeit generally low compared to the general population [56–58].

Interventions aimed at combating vaccine hesitancy should be anchored on contextual issues, as socioeconomic factors seem to have insignificant effects on the COVID-19 vaccine acceptance rates [20]. For example, countries with minimal access to vaccines, like Ecuador and Malaysia had over 90% vaccine acceptance rates [59, 60], whereas countries like France U.S with relatively better access to vaccines had lower COVID-19 acceptance rates [61]. This underscores the need for cautious interpretation of research findings from different settings.

Nearly half (47.3%) of the respondents in our study reported at least one reason for vaccine hesitancy about the safety of vaccines (Fig. 2). Concerns about the safety of COVID-19 vaccines remain a major barrier to vaccine acceptance as previously reported [62, 63]. Other reasons included the inaccessibility of vaccines (12.4%) and individual trust in innate or acquired immunity (10.1%). Barriers to achieving equitable access to vaccines significantly differ between high and low-income countries [64]. This is rather a systemic problem and not intrinsic to the individual populations. Our study like other published reports shows that individual trust in one’s immunity was a common reason for vaccine hesitancy [65]. As expected from the health belief model, a lower perceived risk of COVID-19 harm is associated with the COVID-19 vaccine [66]. It is therefore imperative that the health authorities introduce new strategies, or revise the existing risk communication strategies as a way to minimize COVID-19 vaccine hesitancy.

We also assessed the effectiveness of a community pharmacy based counseling and communication intervention. The study intervention increased the COVID-19 vaccine acceptance rate by 25.4 percent point, from 43.9% to 69.3% (Fig. 3). This finding is evidenced by the significant attitudinal change regarding COVID-19 disease, vaccination exercise, and vaccines (*p*>0.05) at the study end (Table 4, 5). This COVID-19 vaccine acceptance and attitudinal outcome in our study is comparable to findings involving similar interventions, that increased COVID-19 vaccine acceptance by 21% [67]. Conversely, the effectiveness of our intervention was lower compared to studies implementing similar educational/communication interventions (84%) [51]. This could be explained as due to contextual differences as the majority of the studies were conducted in English speaking high-income countries. As proposed by *Bates et al*., the adoption of context-relevant counseling and communication is necessary to reinforce the effect of mass sensitization campaigns, due to the diversity of reasons or barriers associated with COVID-19 vaccine hesitancy [68]. There are numerous structural and systematic barriers to implementing effective public-private partnerships in resource-limited settings like, Uganda; however, this seems to be the way to go to control both current and future public health crises.

Although age has generally been identified as a key predictor of COVID-19 vaccine hesitancy [69, 70], there have been contradicting findings in understanding the age category associated with COVID-19 hesitancy. However, contrary to our findings, a study by *Soares et al.*, indicates that younger age was associated with vaccine hesitancy [71]. COVID-19 risk perception may greatly vary across different age groups in different settings. Therefore, interventions should consider contextual factors influencing different age groups as far as COVID-19 vaccine hesitancy is concerned. The finding that Pentecostal Christians were more likely to be vaccine-hesitant than other socio-demographic categories (Table 3), is consistent with a survey done in the US and Malaysia, where Christian nationalism was a strong predictor for COVID-19 vaccine hesitancy [72, 73]. Pentecostal Christians are a diverse Christian group with a unique functional and structural organization compared to traditional Christianity like Catholicism and Anglicanism. In our setting, there is need for further research to establish the most effective way to communicate and publicize government-led public health interventions among Pentecostal Christian church systems. Participants who attained a primary education only, were less likely to be vaccine-hesitant (Table 3). Although some studies link low literacy with COVID-19 vaccine hesitancy [40], this finding showed the contrary. Participants resident more than 1 km from a public health facility had higher odds of being hesitant to receive COVID-19 vaccines, compared to those who lived near a public health facility (Table 3). The finding that participants who have had a friend or relative diagnosed with COVID-19 were likely to be hesitant to COVID-19 vaccines should be further interrogated; we postulate that COVID-19 disease outcomes of the friend or relative could strongly correlate to one’s decision, whether to vaccinate or not.

As in previous studies [74], having a personal suspicion to have been infected with COVID-19 was associated with greater odds of being hesitant to receive a COVID-19 vaccine. As long as there is the absence of clinical or life-limiting symptoms associated with the ‘suspected’ COVID-19 disease, one may not appreciate the need to vaccinate.

### Study strength and limitations

One of the major strengths of this study was that it involved a four-week follow-up period. Previous studies especially in our setting were largely cross-sectional. Being a prospective follow-up intervention allowed us to capture the true attitudinal change and the resultant decision to receive a vaccine within four weeks. Another strength was that the study would be generalizable to low-resource settings with similar challenges of vaccine accessibility and vaccine hesitancy. Lastly, to minimize the several potential biases, participants were informed that this was research and not part of the pharmacy services. Research assistants were trained and supervised to ensure adherence to the questionnaire to avoid question order biases. Additionally, the investigators were present at the study site at all times to ensure professional conduct and responses to the participant’s concerns. Using the collapsed 3-point Likert scale as compared to the 5-point scale, minimized occurrence of extreme response bias.

Several limitations were present in our study. First, the acceptance to receive COVID-19 vaccine was based on self-reports; implying that these responses may have been influenced by social desirability bias. Secondly, the COVID-19 vaccines were not available at the time the study, thus the participant responses may have been biased as they were not expecting actual vaccination. Lastly, the perceived risk of COVID-19 was generally low at the time of the study; probably affecting the respondents’ attitudes towards COVID-19, vaccines and vaccination programs.

### Generalizability of results

Despite of the study limitations, the findings of the current study can be considered generalizable depending on study context and considering the strengths discussed earlier. Thus, our findings can inform future strategies in designing community pharmacy based counseling programs for vaccination campaigns.

### Sensitivity analysis

No sensitivity analysis was conducted in this study. However, a step-wise backward logistic regression analysis was done to obtain the final logistic regression model.

## Conclusion

COVID-19 vaccine hesitancy is a big challenge in Uganda. This study found that a combination of sociodemographic factors and COVID-19 vaccine perceptions are key predictors of COVID-19 vaccine hesitancy. Structured counseling interventions can improve COVID-19 vaccine acceptance rates. Larger prospective studies should evaluate the effectiveness of similar interventions in community pharmacies and other health care settings.

## Data Availability

The datasets used and/or analyzed during the current study available from the corresponding author on reasonable request.

## Abbreviations

COVID-19: Coronavirus Disease 2019
SARS-CoV-2: Severe Acute Respiratory syndrome coronavirus 2
MGH: Massachusetts General Hospital
MUST: Mbarara University of Science and Technology
SMS: Short Message Services
WHO: World Health Organization
